# Causal association of gastroesophageal reflux disease on irritable bowel syndrome: a two-sample Mendelian randomization study

**DOI:** 10.3389/fgene.2024.1328327

**Published:** 2024-03-27

**Authors:** Huihuan Wu, Jingwei Li, FeiFei Li, Weijian Lun

**Affiliations:** ^1^ Department of Gastroenterology, The Sixth Affiliated Hospital, School of Medicine, South China University of Technology, Foshan, Guangdong, China; ^2^ Department of Gastroenterology, Guangdong Provincial People’s Hospital (Guangdong Academy of Medical Sciences), Southern Medical University, Guangzhou, China; ^3^ Department of Cardiology, Guangdong Provincial Hospital of Chinese Medicine, The Second Affiliated Hospital of Guangzhou University of Chinese Medicine, Guangzhou, Guangdong, China

**Keywords:** gastroesophageal reflux disease, irritable bowel syndrome, Mendelian randomization, causal association, clinical guidance

## Abstract

**Background:**

Recently, observational studies have reported that gastroesophageal reflux disease (GERD) is commonly associated with irritable bowel syndrome (IBS), but the causal relationship is unclear.

**Methods:**

We conducted a two-sample Mendelian randomization study using summary data from genome-wide association studies (GWASs) to explore a causal relationship between GERD (N cases = 129,080) and IBS (N cases = 4,605) of European ancestry. Furthermore, the inverse-variance weighted (IVW) method and a series of sensitivity analyses were used to assess the accuracy and confidence of our results.

**Results:**

We found a significant association of GERD with IBS (NSNP = 74; OR: 1.375; 95% CI: 1.164–1.624; *p* < 0.001). Reverse MR analysis showed no evidence of a causal association for IBS with GERD (NSNP = 6; OR: 0.996; 95% CI: 0.960–1.034; *p* = 0.845).

**Conclusion:**

This study provides evidence that the presence of GERD increases the risk of developing IBS, and it is observed from the reverse MR results that IBS did not increase the risk of GERD.

## 1 Introduction

Gastroesophageal reflux disease (GERD) is one of the most common diseases of the digestive tract and is characterized by heartburn and reflux symptoms caused by reflux of stomach contents. It is estimated that approximately 20% of adults in the West suffer from GERD (Maret-Ouda et al., 2020; Mehta et al., 2021). Irritable bowel syndrome (IBS) is a chronic intestinal dysfunction characterized by altered intestinal function (frequency and/or consistency) and abdominal pain associated with intestinal function (Ford et al., 2017; Camilleri, 2021), with a prevalence of approximately 7%–16% of population in the United States suffering from IBS(Sperber et al., 2021). Both GERD and IBS have a significant financial impact on healthcare systems and have a negative impact on the quality of life (Black and Ford, 2020; Katzka and Kahrilas, 2020; Sperber et al., 2021).

Evidence from observational studies indicates that there is a degree of overlap between GERD and IBS patients (Monnikes et al., 2011; Plaidum et al., 2022), which indicates that GERD and IBS may share a common pathophysiological phenotype on the basis of pathogenic mechanisms (Gasiorowska et al., 2009). Furthermore, several systematic review studies have shown that patients diagnosed with GERD are at a significantly increased risk of being subsequently diagnosed with IBS, according to Lovell’s research, and the pooled OR for GERD in patients with IBS compared with patients without IBS was 4.17 (95% CI, 2.85–6.09). Similarly, patients with a GERD diagnosis were also at a significantly increased risk of IBS (El-Serag et al., 2009; Lovell and Ford, 2012). A randomized clinical trial study showed that in patients with overlapping GERD and IBS, there was a significant improvement in pre-existing IBS symptoms after proton pump inhibitor (PPI) treatment for GERD (Monnikes et al., 2012). However, single cross-sectional designs are frequently used in these observational studies, and there is uncertainty in the results because they are susceptible to potential bias from reverse causality and confounding factors. So far, few studies have investigated the causal relationship between GERD and IBS.

Mendelian randomization (MR) is a genetic instrumental variable approach that employs a single-nucleotide polymorphism (SNP) as an instrument variable (IV) to infer a causal association between two traits, and it has the advantage of reducing bias owing to reverse causality and confounding factors (Ziegler et al., 2015; Davies et al., 2018; Lord et al., 2021). The two-sample MR analysis is a statistical method based on the natural random allocation of genetic variations in evaluating a causal relationship within the summary statistics of GWASs. Therefore, we used a two-sample MR method to comprehensively assess the causal relationship between GERD and IBS.

## 2 Methods

### 2.1 Study design

We performed a two-sample MR analysis to investigate the causal effects of GERD and IBS, and the flow diagram of the study design is shown in Supplementary Figure S1. The forward MR analysis considered GERD as the exposure and IBS as the outcome, while the reverse MR analysis considered IBS as the exposure and GERD as the outcome. Because our research relied on summary statistics from previously published studies, no additional ethical approval or informed permission was required.

### 2.2 Data sources

In this research, the summary statistics of GERD included 129,080 cases and 473,524 controls from a large-scale GWAS meta-analysis (Ong et al., 2022). IBS data were obtained from the FinnGen database, which included a total of 4,605 cases and 182,423 controls (Kurki et al., 2023) (Supplementary Table S1). All the summary statistics for GERD data and IBS data can be downloaded from the Integrative Epidemiology Unit (IEU) Open GWAS database (https://gwas.mrcieu.ac.uk/). All participants in the original study were from European populations.

### 2.3 Instrument selection

Similar to the majority of MR studies, SNPs associated with GERD were selected at a threshold of *p* values <5 × 10^−8^. Because of the limited number of SNPs meeting genome-wide significance in European populations, we used SNPs with a wide threshold (*p* < 5 × 10^−6^) as potential IVs for IBS in European populations for reverse casual MR analysis. To minimize the effect of linkage disequilibrium (LD), SNPs apply strict selection criteria, such as *R* ^2^ = 0.001 with a genetic window of 10,000 kb in the European 1000 Genome reference panel. *F* statistics for each SNP was calculated with the formula *F* = β^2^/SE^2^, in which *F* value < 10 was excluded (Burgess et al., 2011; Burgess and Thompson, 2011).

### 2.4 MR analysis

To investigate the causal relationship between GERD and IBS, we carried out a two-sample MR analysis employing the inverse-variance weighted (IVW) method as the primary method, and *p* < 0.05 was defined as statistically significant (Burgess et al., 2013). Complementary analyses were conducted employing weighted median, MR-Egger regression, and weighted-mode methods, which provided more credible evidence of causality (Bowden et al., 2015; Bowden et al., 2016). The Wald ratio approach was used to calculate the effect estimate of the association between the selected exposure and the outcome of each SNP. All results were presented as the odds ratio (OR) and 95% confidence interval (95% CI).

### 2.5 Sensitivity analyses

We conducted sensitivity analyses to calculate the heterogeneity and pleiotropy in the study, as well as any potential genetic outliers, in order to provide a more credible assessment. The heterogeneity was assessed using MR-Egger and IVW; *p* ≥ 0.05 implies that there is no heterogeneity in the causal relationship. MR-PRESSO outlier analysis and MR-Egger intercept estimate were used to evaluate the horizontal pleiotropy (Verbanck et al., 2018). The leave-one-out analysis removed each SNP from the investigation, and a two-sample MR analysis was conducted using the remaining SNPs as IVs to determine if the whole causal relationship was triggered by a single SNP (Burgess and Thompson, 2017).

### 2.6 Statistical analyses

R software (version 4.3.0) was employed for all statistical analyses. The R-based package “Two Sample MR” was used to perform the MR analysis (Hemani et al., 2018). *p* values were normally two-sided, and *p* < 0.05 was defined as statistically significant.

## 3 Results

### 3.1 Genetic instrumental variables

After removing linkage disequilibrium and palindromes, we obtained 74 independent SNPs for GERD and six independent SNPs for IBS. Moreover, the *F*-statistic of 74 GERD-associated SNPs (the median *F*-statistic was 35.155, ranging from 29.749 to 96.029) and six IBS-associated SNPs (the median *F*-statistic was 21.259, ranging from 21.231 to 23.514) was >10 (Supplementary Table S2), which suggests that the causality results obtained in our study can be interpreted without regard to weak instrumental variables. The comprehensive information of the selected SNPs is shown in forest plots (Supplementary Figure S1A, B) and listed in Supplementary Table S3.

### 3.2 Causal effect of GERD on the risk of IBS

**FIGURE 1:**
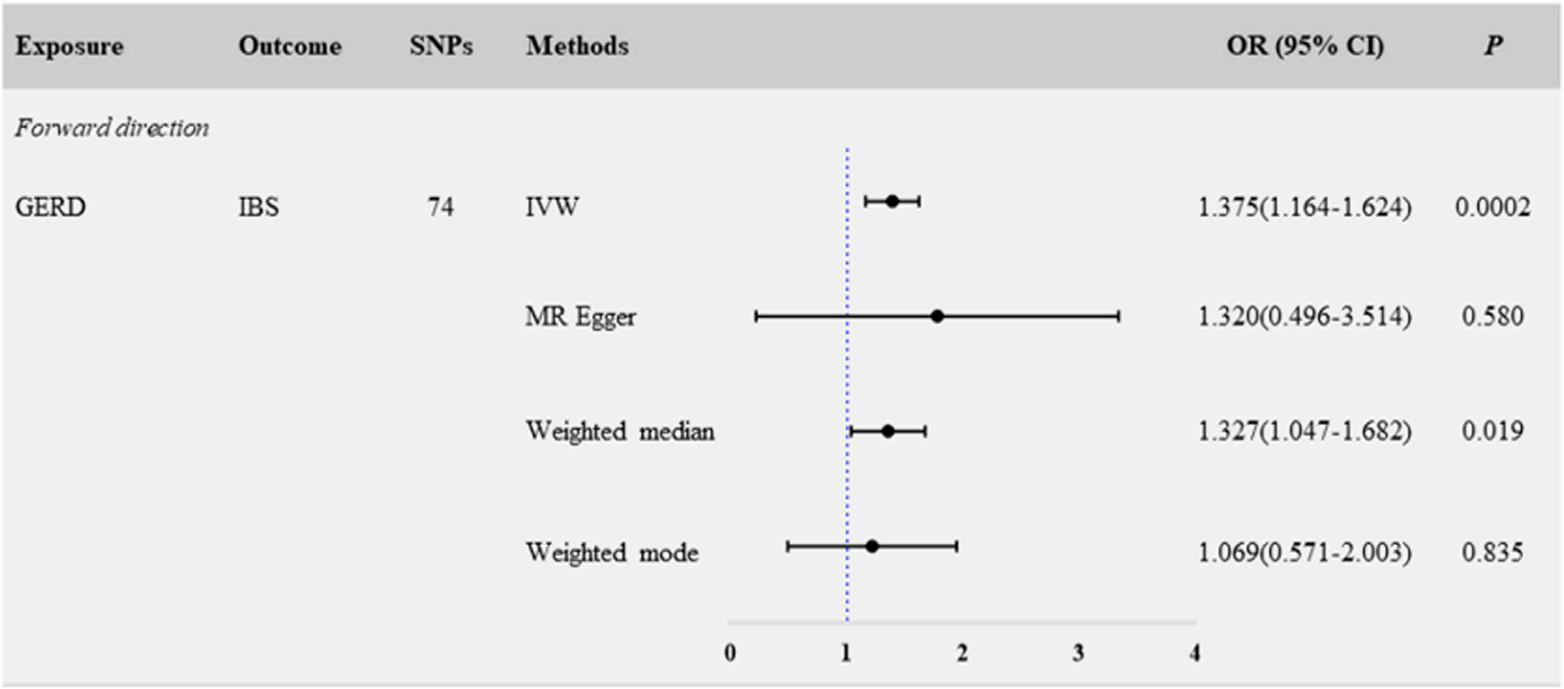
Forward MR analysis: causal estimates for the effect of GERD and IBS.

In the primary analyses using IVW, the results showed that genetically predicted GERD was positively related to IBS (N_SNP_ = 74; OR: 1.375; 95% CI: 1.164–1.624; *p* < 0.001) (Figure 1; Figure 3A). Consistent causal effect estimates of GERD on the risk of IBS were observed using the weighted-median regression method (N_SNP_ = 74; OR: 1.327; 95% CI: 1.047–1.682; *p* = 0.019) (Figure 1; Figure 3A).

We next performed a sensitivity analysis to assess the robustness of the causal relationship between GERD and IBS. The Egger-intercept of the MR-Egger analysis result (Table 1, intercept = 0.001, *p* = 0.934) and the MR PRESSO result (Table 1, global test *p* = 0.806) showed no evidence of potential directional pleiotropy. In addition, combining of Cochran’s Q *p*-value in IVW (Table 1, Q = 62.633, *p* = 0.801) and MR-Egger (Table 1, Q = 62.626, *p* = 0.777) methods with the funnel plot (Supplementary Figure S2A) indicated that the causal relationship between GERD and IBS was without heterogeneity. We also systematically excluded individual SNPs and repeated the MR analysis sequentially; no potential driving SNPs for the evaluated causal relationship between GERD and IBS were observed in the leave-one-out analysis (Supplementary Figure S3A). These findings showed that GERD in patients is a causal risk factor for the development of IBS.

**TABLE 1:** Pleiotropy and heterogeneity analysis between gastroesophageal reflux disease and irritable bowel syndrome.

Exposure	Outcome	MR-Egger		MR PRESSO	Heterogeneity		
		Intercept	*p*	Global test *p*	Method	Q	Q_*pval*
Forward direction							
GERD	IBS	0.001	0.934	0.806	IVW	62.633	0.801
	—	—	—	—	MR Egger	62.626	0.777
Reverse direction							
IBS	GERD	0.0005	0.972	0.993	IVW	0.322	0.997
	—	—	—	—	MR Egger	0.321	0.988

GERD, gastroesophageal reflux disease; IBS, irritable bowel syndrome; MR PRESSO, Mendelian Randomization Pleiotropy RESidual Sum and Outlier; IVW, inverse-variance weighted.

### 3.3 No causal effect of IBS on GERD

**FIGURE 2:**
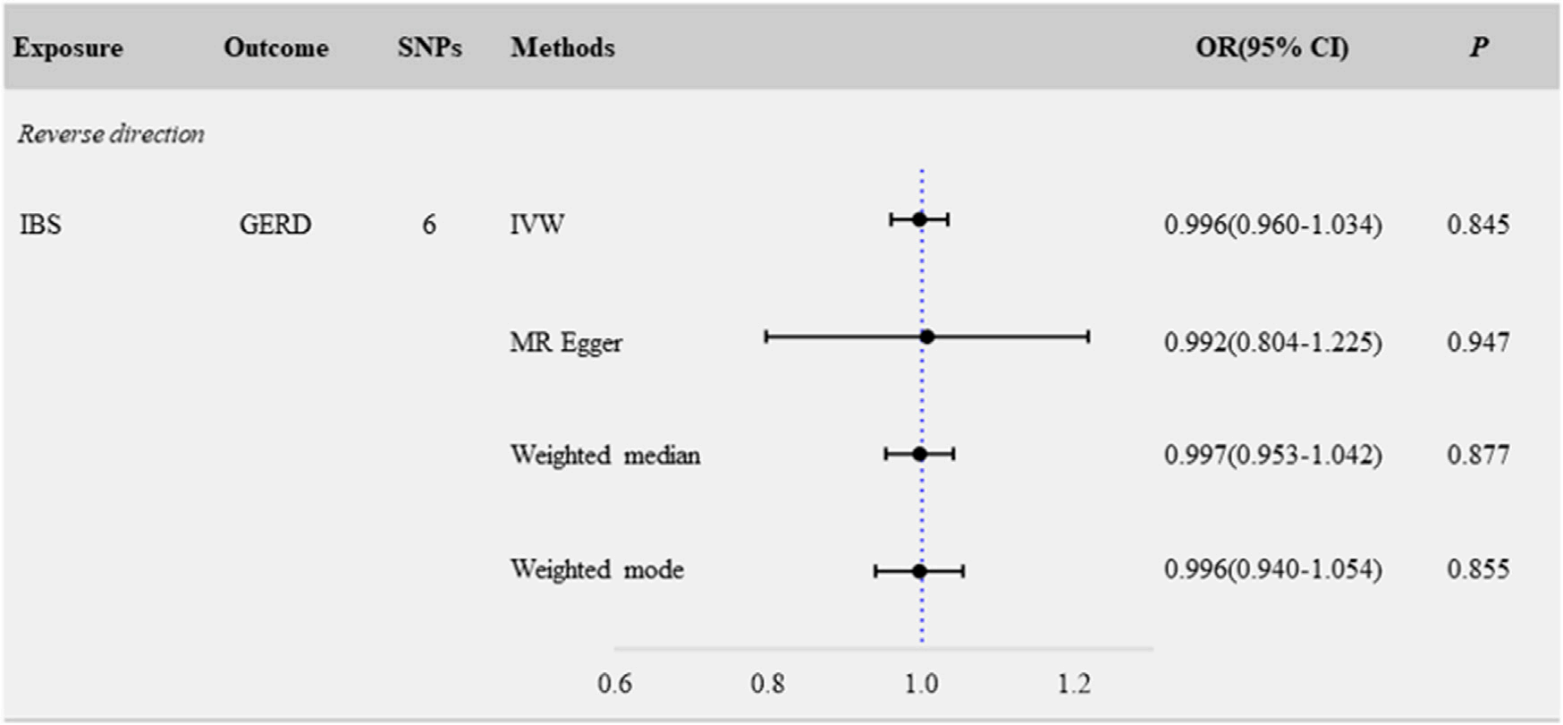
Reverse MR analysis: causal estimates for the effect of IBS and GERD.

**FIGURE 3:**
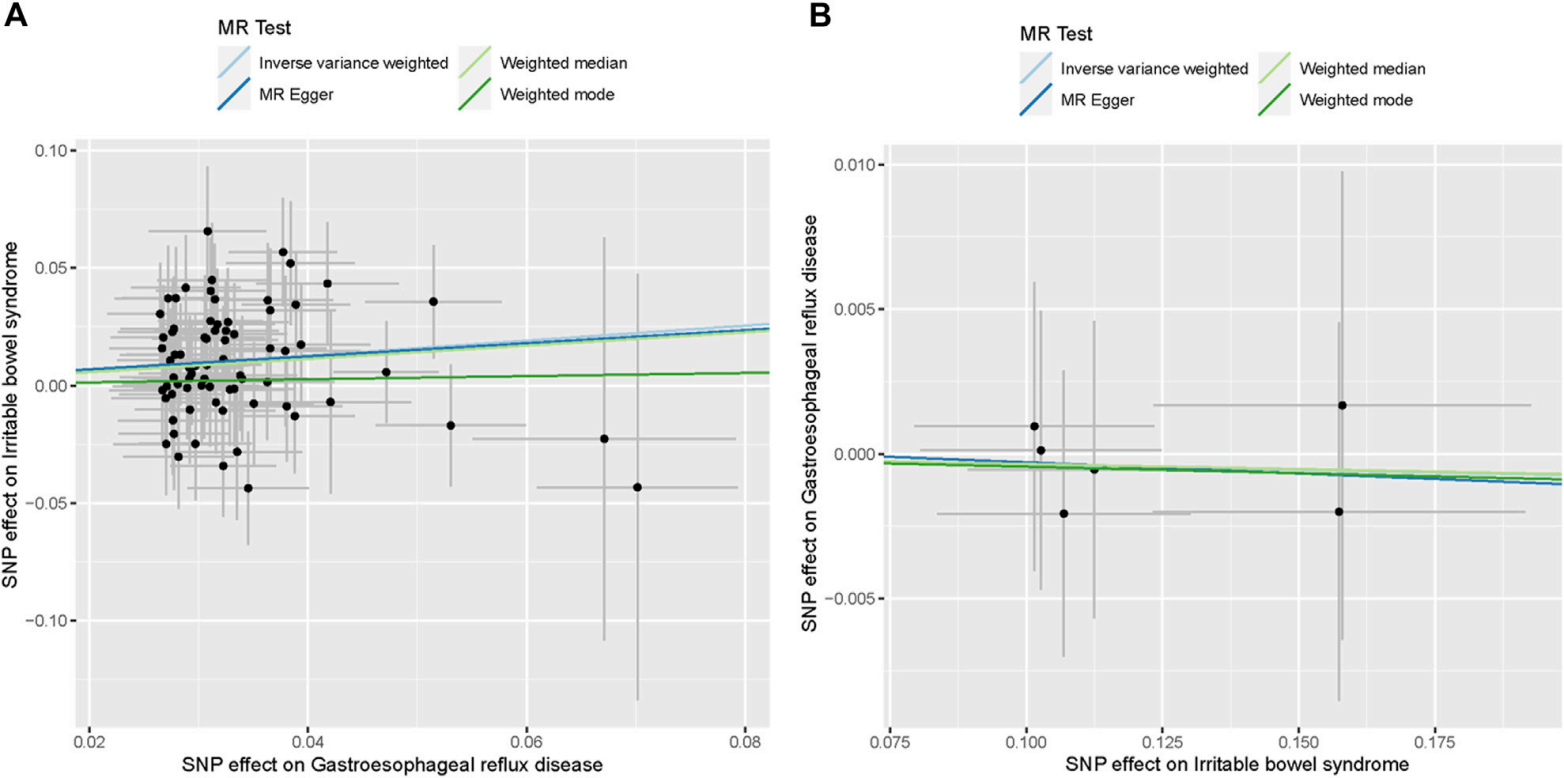
Scatter plots of the causal association between GERD and IBS. **(A)** Scatter plot of the causal association between GERD and IBS. **(B)** Scatter plot of the causal association between IBS and GERD.

We evaluated the causal associations of IBS on GERD by IVW, and the result revealed that IBS was not causally relevant to critical GERD (*N*_SNP_ = 6; OR: 0.996; 95% CI: 0.960–1.034; *p* = 0.845) (Figure 2, Figure 3B). No significant association was found in the other methods, including MR Egger (*N*_SNP_ = 6; OR: 0.992; 95% CI: 0.804–1.225; *p* = 0.947), weighted-median (*N*_SNP_ = 6; OR: 0.997; 95% CI: 0.953–1.042; *p* = 0.877), and weighted-mode (*N*_SNP_ = 6; OR: 0.996; 95% CI: 0.940–1.054; *p* = 0.855) (Figure 2, Figure 3B, Supplementary Figure S2B).

Sensitivity analyses were performed to evaluate the robustness of the causal association between IBS and GERD. The Egger-intercept of the MR-Egger analysis result (Table 1, intercept = 0.0005, *p* = 0.972) and the MR PRESSO result (Table 1, global test *p* = 0.993) showed no evidence of potential directional pleiotropy. Assessment of Cochran’s Q *p*-value in IVW (Table 1, Q = 0.322, *p* = 0.997) and MR-Egger (Table 1, Q = 0.321, *p* = 0.988) methods indicated no heterogeneity. Furthermore, when any single SNP was removed, the observed associations were not altered notably in the leave-one-out analysis (Supplementary Figure S3B).

## 4 Discussion

In general, our findings provide relatively strong evidence that GERD patients might contribute to the development and advancement of IBS when compared to healthy individuals. However, the reverse MR analysis showed no evidence for the causal relationship of IBS on GERD. Furthermore, we performed a sensitivity analysis on the findings mentioned above, and the results of the causal relationship were consistent and robust, and *vice versa*. Therefore, our findings indicate a complex genetic interaction between GERD and IBS and suggest that IBS could be caused by specific pathogenic mechanisms observed in GERD.

Previous studies have extensively reported the frequent co-occurrence of GERD and IBS (de Bortoli et al., 2013; Lacy et al., 2019; Lei et al., 2019; Nwokediuko et al., 2020; Patcharatrakul et al., 2021). A systematic review and meta-analysis demonstrated a high degree of overlap between GERD and IBS (Eusebi et al., 2018). Specifically, a single-center cross-sectional study using data from 168 IBS patients aged 18–60 in southern Iran found that IBS was associated with a higher risk of GERD (Gholamnezhad et al., 2023). Similarly, in a comparative study in Taiwan including 273 GERD patients, it was discovered that GERD was associated with an increased risk of IBS (Lei et al., 2019). Furthermore, a recent MR study has observed a causal association between GERD and IBS, which supports our findings to a certain extent (Li et al., 2023). However, in their study, patients with IBS had an increased risk of developing GERD, which is inconsistent with our findings. In our study, the risk of IBS was increased in patients with GERD, but in patients with existing IBS, no increased risk of GERD was observed. In contrast to previous studies, we have evaluated the causal relationship between GERD and IBS by leveraging the latest GWAS summary data, and there was no sample overlap between exposures and outcomes, which enhances evidence to assessment of causality between IBS and GERD disorders, and our study also conducted reverse validation to assist in clarifying the direction of the relationship. In addition, we validate our results by employing a variety of methods and a series of sensitivity analyses to make the results more reliable.

In patients with IBS, the frequent coexistence of features, or comorbidity, with GERD may underlie a common genetic cause and/or pathogenic mechanism between the two disorders. It is well known that although IBS is considered to have no pathological features, its pathogenesis is clearly related to motor (transport) disorders and local visceral hypersensitivity (Holtmann et al., 2016; Simren et al., 2019). It has been postulated that motor (transport) disorders and local visceral hypersensitivity are associated with GERD patients (Yoshida et al., 2013; Ustaoglu and Woodland, 2023). In addition, prior studies reported that GERD and IBS have found genetic overlaps with the established risk factors with depression (Wu et al., 2021). Diet plays an important role. Several randomized controlled studies on both GERD and IBS have reported the benefits of a low-FODMAP diet on gastrointestinal symptoms and the quality of life in patients with IBS (McIntosh et al., 2017; Wilson et al., 2020). A recent study has demonstrated that the symptoms of GERD and gastric reflux are aggravated by a high-FODMAP diet (Plaidum et al., 2022). It is critical that we investigate the mechanisms of GERD and IBS because we do not yet understand the inherited genetic makeup or common etiology of these conditions. Our findings clarify the causal relationship between GERD and IBS, which should encourage clinicians to pay more attention to the clinical manifestations of IBS in patients with GERD and provide clinical strategies for early detection and treatment of IBS.

The main advantage of this study is that we employed a bidirectional Mendelian randomization analysis method for the first time and confirmed the positive causal relationship of GERD and IBS. Compared with previous observational studies, the application of Mendelian randomization assists in reducing reverse causation bias and residual confounding encountered in observational studies. In addition, we found no heterogeneity and multiple effects influencing the outcomes between GERD and IBS, which properly avoided the interference of negative results and supports the accuracy of our findings. However, there are some limitations to our study. First, only people with European ancestry were included in our study, and our findings may not be generalized to other races and ethnicities. Second, since sex-stratified GWAS data were not available, we were unable to perform a deeper analysis to investigate the specific subpopulation, such as sex affection between two diseases. The availability of high-quality GWASs focused on the specific sex subgroup of GERD or IBS in the future would be valuable. In addition, although the results of all methods are robust, a bias could still exist due to a pool of genetic instruments, a small sample size, or probable sample overlap between exposure and the outcome.

## 5 Conclusion

Taken together, our bidirectional MR study demonstrated that patients with GERD were more likely to develop IBS, but the patients with IBS were less likely to develop GERD. To better prevent IBS, it is recommended that clinicians pay more attention to whether individuals with GERD have clinical manifestations of IBS. Moreover, it is necessary to conduct additional research on the pathophysiological mechanisms underlying the interaction between GERD and IBS.

## Data Availability

The datasets presented in this study can be found in online repositories. The names of the repository/repositories and accession number(s) can be found in the article/Supplementary Material.
