# Supplementary material for: Causal association of gastroesophageal reflux disease on irritable bowel syndrome: a two-sample Mendelian randomization study
Source: Front Genet. 2024 Mar 27;15:1328327. doi: 10.3389/fgene.2024.1328327 (PMC11004226; doi:10.3389/fgene.2024.1328327)
Supplement: Supplementary file 1 [file DataSheet1.PDF]

Supplementary Information

Supplementary Figure 1 Three rigorous assumptions underpin the principle and validity of MR analysis. The genetic instruments should be (1) associated with the GERD, (2) affected IBS only through the GERD, and (3) independent of any confounders in the relation between GERD and IBS.

Supplementary Figure S1. The individual SNP final selected for these causal results were displayed in forest plots. (A) The individual SNP final selected for the effect of GERD on IBS. (B) The individual SNP final selected for the effect of IBS on GERD.

Supplementary Figure S2. Funnel plot was used to detect whether the causal association between GERD on IBS was along with obvious heterogeneity and vice versa (A) GERD on IBS (Forward direction); (B) IBS on GERD (Reverse direction).

Supplementary Figure S3. Leave-one-out analysis plots. (A) Leave-one-out plot of MR analyses to assess whether any individual SNP was driving the causal effect of GERD on IBS (Forward direction). (B)Leave-one-out plot of MR analyses to assess whether any individual SNP was driving the causal effect of IBS on GERD.

Supplementary Table S1. Sources of the GWAS datasets utilized in our study.

Supplementary Table S2. Summary of Genetic Instruments identified for MR Analyses.

Supplementary Table S3. Characteristics of the single-nucleotide polymorphisms used as instrumental variables.

Supplementary Figure 1

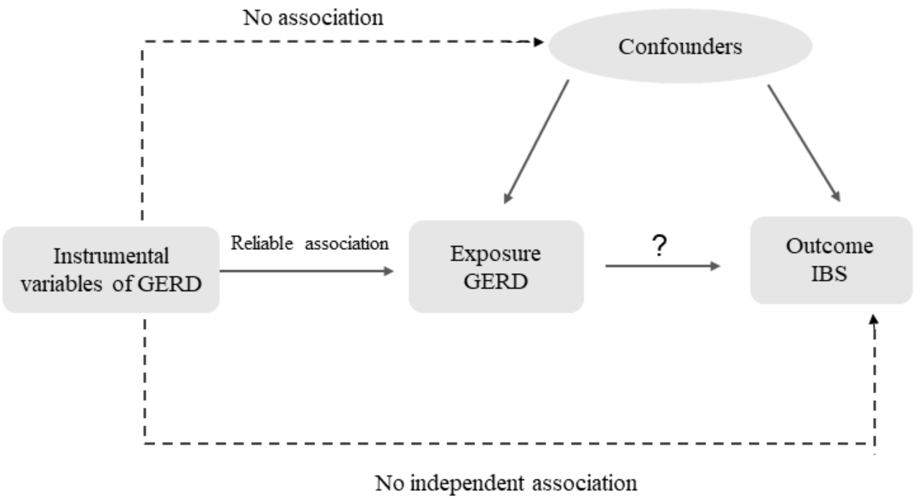

Supplementary Figure S1

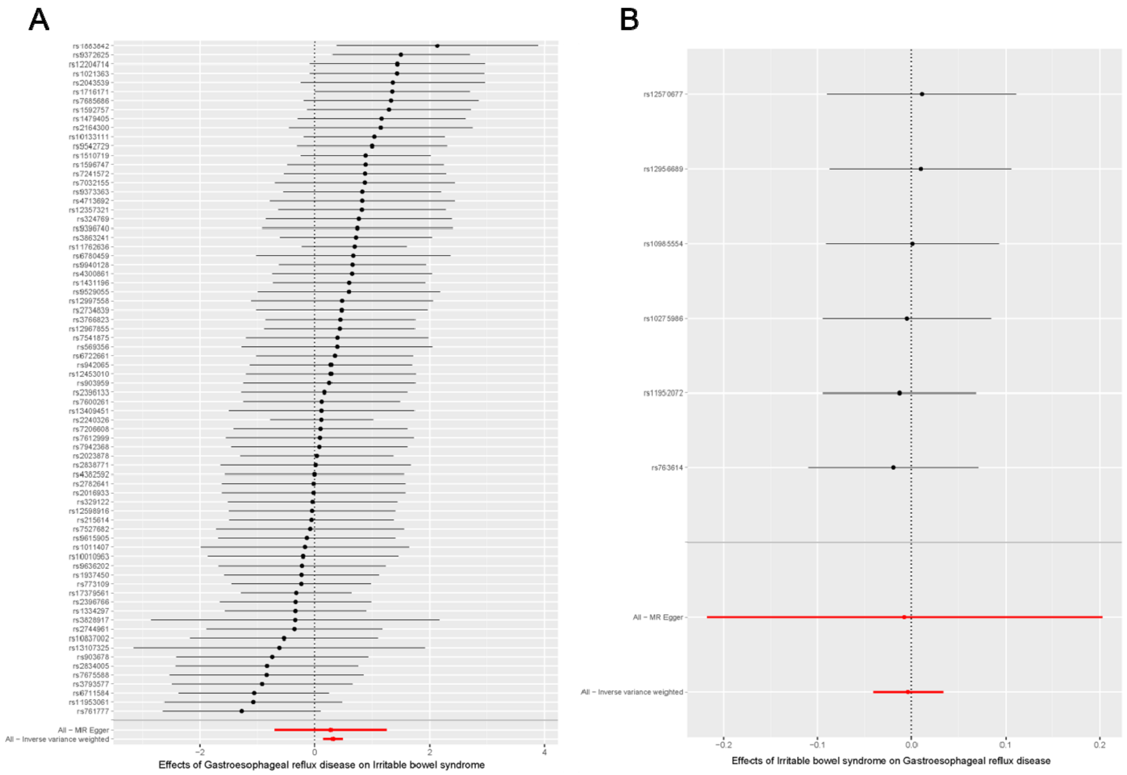

Supplementary Figure S2

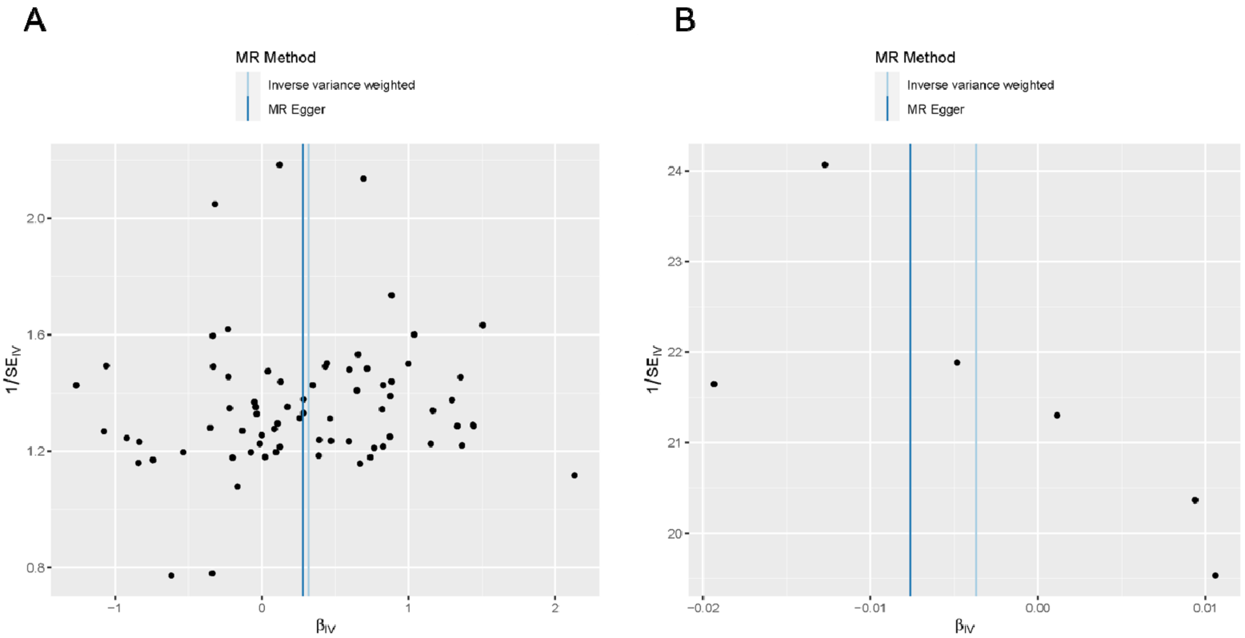

Supplementary Figure S3

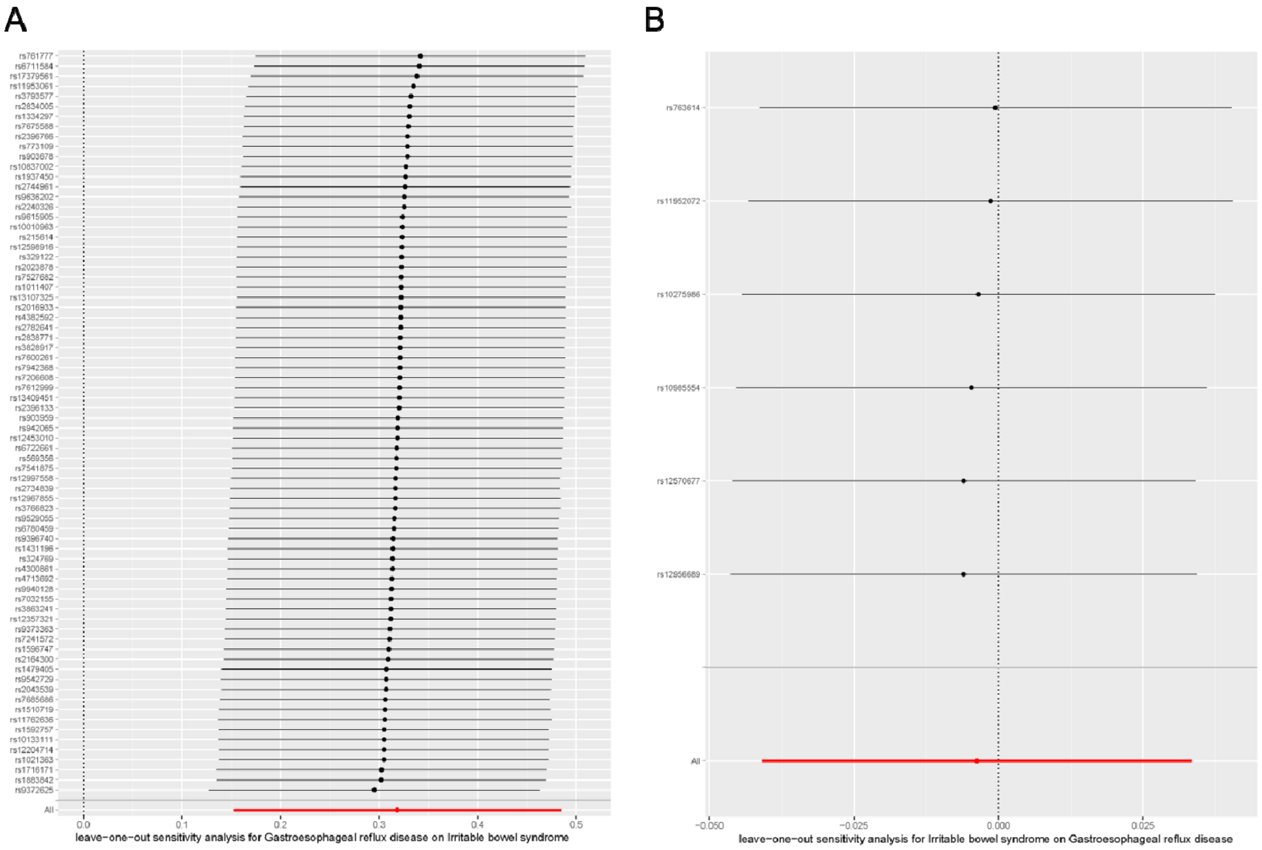

Supplementary Table S1. Sources of the GWAS datasets utilized in our study.

Traits	Year	Data sources	Ancestry	Sample size (Ncase/Ncontrol)	Number of SNPs
GERD	2021	ebi-a-GCST90000514	European	129,080/473,524	16,380,376
IBS	2021	finn-b-K11_IBS	European	46,05/182,423	2,320,781

GERD, Gastroesophageal reflux disease; IBS, Irritable bowel syndrome; SNPs, single-nucleotide polymorphisms

Supplementary Table S2 Summary of Genetic Instruments identified for MR Analyses.

Exposure	Significant level	No. of SNPs	F statistics*
GERD	5e-08	74	35.155(29.749-96.029)
IBS	5E-6	6	21.259(21.231-23.514)

* Median and range (minimum and maximum). GERD, Gastroesophageal reflux disease; IBS, Irritable bowel syndrome; SNPs, single-nucleotide polymorphisms

Supplementary Table S3. Characteristics of the single-nucleotide polymorphisms used as instrumental variables.

SNP	Chr	Location	EA	OA	Association with IBS		
					β	SE	*P*
rs10010963	4	159839313	T	C	-0.2	0.849	0.814
rs1011407	2	60665768	G	A	-0.166	0.927	0.858
rs10133111	14	103377321	A	G	1.039	0.625	0.096
rs1021363	10	106610839	G	A	1.438	0.775	0.064
rs10837002	11	38565727	G	C	-0.535	0.835	0.522
rs11762636	7	2061111	A	C	0.693	0.468	0.139
rs11953061	5	120144025	T	C	-1.076	0.788	0.172
rs12204714	6	152235339	T	C	1.444	0.777	0.063
rs12357321	10	21790476	A	G	0.82	0.744	0.271
rs12453010	17	50316131	T	C	0.283	0.751	0.706
rs12598916	16	60658751	G	C	-0.042	0.74	0.955
rs12967855	18	35138245	G	A	0.432	0.67	0.519
rs12997558	2	41704580	A	G	0.471	0.809	0.56
rs13107325	4	103188709	T	C	-0.617	1.293	0.633
rs1334297	13	58335375	A	G	-0.335	0.626	0.593
rs13409451	2	144257639	G	A	0.123	0.823	0.881
rs1431196	18	50832102	G	A	0.595	0.676	0.378
rs1479405	12	15387519	T	C	1.166	0.746	0.118
rs1510719	4	140938116	C	T	0.885	0.576	0.125
rs1592757	5	103889998	C	G	1.296	0.727	0.075

rs1596747	2	193802478	G	A	0.885	0.695	0.203
rs1716171	12	123716376	T	C	1.354	0.688	0.049
rs17379561	1	98340139	T	A	-0.32	0.488	0.512
rs1883842	20	41223062	G	T	2.131	0.895	0.017
rs1937450	1	66478840	G	T	-0.228	0.687	0.74
rs2016933	3	65653157	G	C	-0.016	0.815	0.984
rs2023878	19	18834124	T	C	0.041	0.678	0.951
rs2043539	7	12253880	A	G	1.364	0.82	0.096
rs215614	7	32347335	A	G	-0.052	0.731	0.944
rs2164300	4	67813017	T	C	1.152	0.816	0.158
rs2240326	3	50128386	A	G	0.121	0.458	0.792
rs2396133	7	109197067	G	A	0.174	0.739	0.814
rs2396766	7	114318071	A	G	-0.332	0.671	0.62
rs2734839	11	113286490	T	C	0.466	0.762	0.541
rs2744961	6	34655000	T	C	-0.353	0.781	0.651
rs2782641	1	44013355	A	G	-0.015	0.816	0.986
rs2834005	21	34291708	C	T	-0.835	0.811	0.303
rs2838771	21	46501576	C	G	0.021	0.847	0.98
rs324769	12	83969240	T	C	0.766	0.826	0.354
rs329122	5	133864599	A	G	-0.035	0.753	0.963
rs3766823	1	32197257	A	G	0.442	0.666	0.507
rs3793577	9	23737627	G	A	-0.921	0.803	0.251
rs3828917	6	31465917	T	G	-0.338	1.28	0.792
rs3863241	8	73890335	T	C	0.717	0.674	0.287
rs4300861	2	22549441	T	C	0.648	0.71	0.361
rs4382592	9	134870755	G	T	0	0.796	1
rs4713692	6	33807638	T	C	0.826	0.822	0.315
rs569356	1	29136686	G	A	0.388	0.844	0.646
rs6711584	2	104421692	A	G	-1.06	0.67	0.113
rs6722661	2	100806588	A	G	0.347	0.701	0.62
rs6780459	3	104624105	T	A	0.668	0.864	0.44
rs7032155	9	122672771	A	C	0.872	0.8	0.276
rs7206608	16	82872628	G	C	0.106	0.772	0.89
rs7241572	18	77580712	A	G	0.875	0.72	0.224
rs7527682	1	189172684	G	A	-0.075	0.836	0.929
rs7541875	1	190957589	G	A	0.391	0.807	0.628
rs7600261	2	212622818	T	C	0.127	0.695	0.855
rs7612999	3	35678337	A	G	0.095	0.835	0.909
rs761777	10	134938075	G	A	-1.265	0.701	0.071
rs7675588	4	80734978	A	C	-0.841	0.862	0.329
rs7685686	4	3207142	G	A	1.332	0.777	0.086
rs773109	12	56374695	A	G	-0.231	0.617	0.708
rs7942368	11	76465362	T	C	0.085	0.783	0.913
rs903678	1	201809918	A	G	-0.743	0.854	0.385

rs903959	8	142630782	A	T	0.257	0.761	0.735
rs9372625	6	98344031	A	G	1.506	0.612	0.014
rs9373363	6	143150043	G	A	0.826	0.701	0.238
rs9396740	6	17023108	A	G	0.74	0.848	0.383
rs942065	14	94032065	A	G	0.283	0.725	0.696
rs9529055	13	66957533	A	G	0.593	0.81	0.464
rs9542729	13	31833578	G	C	0.999	0.666	0.134
rs9615905	22	48875699	T	C	-0.134	0.787	0.865
rs9636202	19	18449238	A	G	-0.22	0.742	0.767
rs9940128	16	53800754	A	G	0.656	0.653	0.315
All - Inverse variance weighted					0.318	0.085	<0.001
All - MR Egger					0.278	0.499	0.58

**SNP**	**Chr**	**Location**	**EA**	**OA**	**Association with GERD**		
					**β**	**SE**	***P***
rs10275986	7	13254927	T	C	-0.005	0.046	0.916
rs10985554	9	124924543	G	A	0.001	0.047	0.981
rs11952072	5	173630504	C	A	-0.013	0.042	0.759
rs12570677	10	129594734	A	T	0.011	0.051	0.836
rs12956689	18	45620634	A	G	0.009	0.049	0.849
rs763614	17	15022096	T	C	-0.019	0.046	0.675
All - Inverse variance weighted					-0.004	0.019	0.845
All - MR Egger					-0.008	0.107	0.947

Chr, chromosome; EA, effect allele; OA, other allele; IBS, Irritable bowel syndrome; GERD, Gastroesophageal reflux disease; SE, standard error; SNP, single-nucleotide polymorphism.
